# Design and Optimization of Dolmen-like Nanoantenna on Silicon Dioxide for Sensing Applications

**DOI:** 10.3390/s26103019

**Published:** 2026-05-11

**Authors:** Hesham A. Attia, Mohamed A. Swillam

**Affiliations:** Department of Physics, School of Science and Engineering, The American University in Cairo, New Cairo 11835, Egypt; hesham_ahmed@aucegypt.edu

**Keywords:** plasmonic resonator, fano resonance, dolmen nanostructure, refractive index sensor, water salinity sensor

## Abstract

We present the development of an infrared sensor based on a meta surface utilizing Dolmen plasmonic nanostructures. This meta surface is engineered to enhance the absorption of infrared light at a specific wavelength. The sensor is optimized for high sensitivity and selectivity in the infrared spectrum. This straightforward meta surface sensor shows promise for various applications, including gas sensing, biosensing, and security. The design is compact and easy to fabricate with studied fabrication tolerance ensuring reliable performance. The sensor was tested for water-based sensing applications, and we tested its performance by using different materials such as ZrN, TiN, Cr, and Au on silicon dioxide. In a separate configuration, a gold nanostructure was fabricated on a silicon layer over a silicon dioxide base to examine the resulting plasmonic response. The results surpass those of other water quality sensors, underscoring the potential of this design for high-performance sensing. The sensor’s high sensitivity and low fabrication costs make it a promising technology for future sensing and monitoring applications.

## 1. Introduction

Saline water comprises about 97% of the Earth’s total water supply, while only a minor fraction is freshwater, located in glaciers, ice caps, groundwater, and surface water bodies. Seawater presents significant potential as a source for drinking water and industrial use, as well as being a crucial provider of essential minerals for human health. It is composed of a complex mixture of ions such as sodium, chloride, magnesium, sulfate, calcium, and bicarbonate, along with dissolved gases, organic matter, and trace elements. The ion concentration in seawater is significantly higher than in freshwater [1]. Oceans and seas play a crucial role in maintaining the stability of the global climate system. They absorb a substantial amount of carbon dioxide emissions from fossil fuel combustion, helping to mitigate the impact of greenhouse gases and contributing to climate stability. Marine ecosystems, which have evolved over millions of years, support a diverse range of species adapted to saline environments. The health benefits of saline water have been acknowledged for centuries, with its use linked to improvements in cardiovascular health, skin conditions, and overall well-being.

Marine environments are also being explored for their potential in renewable energy production, with technologies such as tidal energy, wave energy, and ocean thermal energy conversion being developed to provide sustainable energy solutions. However, pollution of marine waters poses significant public health risks, especially in developing regions where contaminated water can lead to outbreaks of waterborne diseases. Therefore, sustainable management and protection of marine resources are essential to ensure their continued availability and the health of both human populations and the broader environment [2].

According to the Soil Resources Development Institute (SRDI), there has been a documented average annual increase of 0.74% in the area affected by salinity intrusion between 1973 and 2009. During this period, the extent of salinity-affected regions expanded significantly, growing from 0.83 million hectares to 1.06 million hectares [3]. This trend highlights the escalating challenge of salinity intrusion, which poses a substantial threat to agricultural productivity, water quality, and ecosystem health in the affected areas.

The contamination of saline water, particularly in urban areas, has become a significant global concern. This issue is exacerbated by factors such as evaporation, industrial activities, and saltwater intrusion. Anthropogenic activities in urban areas contribute to the alteration of natural water salinity levels. To measure seawater salinity, a system that assesses conductivity, temperature, and depth (CTD) is typically used. However, this method has limitations. The electro salinity sensors in this system rely on the correlation between electrical conductivity and the concentration of chloride ions in the water [4]. The salinity of a solution significantly affects its refractive index (RI), a principle that has been leveraged in the development of various optical fiber sensor-based methods for salinity measurement. These methods have been rigorously tested and refined to enhance accuracy and reliability. Among the prominent techniques are the optical refraction method, which utilizes a refractometer or an electromagnetic resonator to ascertain the refractive index [5,6,7].

Another advanced technique is the optical fiber grating method [8,9], which employs fiber Bragg gratings (FBGs) or long-period gratings (LPGs) to detect shifts in the refractive index. This method benefits from the high sensitivity and specificity of grating-based sensors, making it suitable for real-time monitoring of salinity in various environments.

The surface plasmon resonance (SPR) method is also widely used for salinity measurement. This technique exploits the resonant oscillation of conduction electrons at the interface between a metal and a dielectric, which is sensitive to changes in the refractive index [10].

SPR sensors are known for their high sensitivity and ability to provide real-time, label-free detection of salinity variations. Recent paradigm shifts in silicon photonics have facilitated the development of all-dielectric metasurfaces designed to circumvent the inherent ohmic dissipation losses associated with noble metals demonstrated the efficacy of such platforms by engineering a silicon-based double Fano resonance for gas detection, reporting a state-of-the-art Figure of Merit (FOM) exceeding 11,000 within the telecommunication C-band [11]. Notwithstanding the high-Quality factors achievable in all-dielectric regimes, these systems often fail to replicate the extreme subwavelength field localization characteristic of plasmonic architectures. Consequently, the implementation of Fano resonances within metal-insulator-metal (MIM) configurations has been established as a cornerstone for enhancing detection resolutions in biochemical sensing [12]. As elucidated by Adhikari et al., the distinctive asymmetric line shape—facilitated by the destructive interference between a discrete resonant state and a broad spectral continuum—yields a significantly heightened sensitivity to local refractive index perturbations relative to conventional Lorentzian profiles. However, conventional MIM-based Fano sensors frequently encounter limitations such as radiative damping and substantial fabrication constraints. In response, hybrid metal-dielectric nanostructures have emerged as a synergistic platform, merging the intense electromagnetic field enhancement of plasmonics with the low-loss dielectric properties of high-index materials. Indeed, it has been evidenced that incorporating a dielectric nanoring into a metallic oligomer can modulate the multiplicity of Fano dips and enhance near-field intensity twenty-fold compared to purely metallic counterparts [13]. Nevertheless, current research in hybrid platforms remains largely confined to the visible spectrum; thus, there is a critical imperative for robust, near-infrared (NIR)-optimized systems that leverage CMOS-compatible materials like silicon to facilitate scalable, high-performance environmental monitoring.

Plasmonic sensors offer several advantages over other sensor types, such as optical fiber and photonic crystal sensors. These advantages include their compact dimensions, rapid response times, and high sensitivity to variations in the refractive index and ambient temperature [14]. Over the past few years, plasmonic sensing has evolved from metal nanostructures to more exotic material systems, and from two-dimensional geometries to multi-dimensional architectures. Hybrid systems involving three dimensional (3D) resonant cavities incorporating silver and metal–organic frameworks (MOFs) have been shown to offer high performance sensing at the molecular level, by combining the advantages of the porous molecular framework for trapping molecular species into the molecular scale and the high density of electromagnetic hot spots within the hybrid cavity for enhancing plasmonic field localization [15]. On the other hand, tuning multi-band absorbers to achieve surface plasmon resonance (SPR) in the terahertz regime using graphene as active material enables us to benefit from the unique properties of 2D material and attain high sensitivity and spectral tunability in a range of frequency where static metallic structures have limitations. While great efforts have been devoted to achieve high sensitivity in the visible range and tunability in the terahertz regime, there is significant potential for further development in near-infrared (NIR) and further into the IR) range, where robust, scalable, and CMOS compatible hybrid systems with high field confinement and detection resolution in aqueous environments are in urgent need [16]. The recently introduced dolmen nanostructure provides an example of such plasmonic nanostructures for NIR sensing with high resolution, while preventing the involvement of molecular species in multiple dimensions of chemical frameworks or active gating.

**Figure 1:**
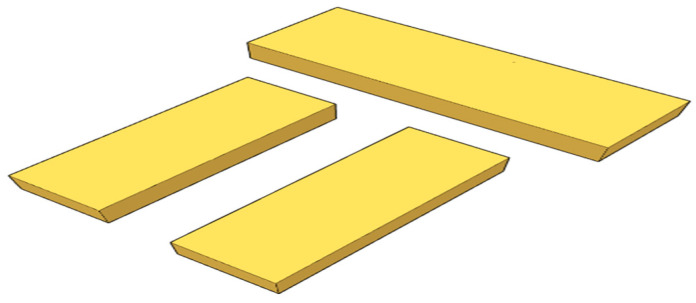
Dolmen nanostructure, which consists of three rectangles that have spaces between them. The top rectangle is called the top dolmen while bottom rectangles are called left and right dolmen.

A dolmen nanostructure, which is composed of three rectangular nanorods separated by gaps, serves as an effective platform for plasmonic sensing applications. This configuration is illustrated in Figure 1 [17]. The unique sensing capabilities of dolmen nanostructures arise from the interaction of two distinct modes, leading to the phenomenon known as Fano resonance.

**Figure 2:**
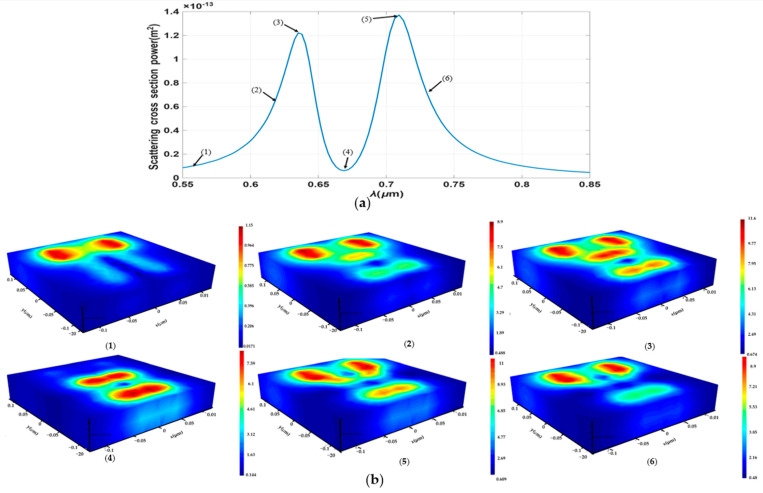
(**a**) The relation between scattering cross section power vs. the wavelength in Dolmen nanostructure. (**b**) The corresponding electric field distribution with different wavelength according to the graph (1) **λ** = 0.55 µm, (2) **λ** = 0.622 µm, (3) **λ** = 0.635 µm, (4) **λ** = 0.669 µm, (5) **λ** = 0.709 µm, (6) **λ** = 0.726 µm.

Fano resonance is characterized by the interference between a narrow, discrete mode, referred to as the “dark mode,” and a broad, continuous mode, known as the “bright mode” [18]. The dark mode is typically a sub radiant mode with a high-quality factor, while the bright mode is a super radiant mode with a lower quality factor. The coupling of these modes results in an asymmetric line shape in the resonance spectrum, which is highly sensitive to changes in the surrounding environment, as shown in Figure 2.

Surface plasmon resonance (SPR) is another critical aspect of plasmonic sensors. SPR occurs when incident light induces collective oscillations of free electrons at the interface between a metal and a dielectric. This phenomenon is highly sensitive to changes in the refractive index near the metal surface, making it an effective mechanism for detecting various analytes [18].

The combination of SPR and Fano resonance in dolmen nanostructures enhances the sensitivity and specificity of the sensor, enabling the detection of minute changes in the local environment. Recent advancements in plasmonic sensor technology have focused on optimizing the structural parameters of dolmen nanostructures to achieve stronger and more tunable Fano resonances. By adjusting the dimensions and spacing of the nanorods, researchers can control the coupling strength between the dark and bright modes, thereby fine-tuning the resonance characteristics [19]. This tunability is crucial for developing sensors with tailored sensitivity and selectivity for specific applications. Moreover, the integration of dolmen nanostructures with other optical components, such as waveguides and photonic crystals, has been explored to further enhance the performance of plasmonic sensors. These hybrid systems leverage the strengths of both plasmonic and photonic technologies, resulting in sensors with improved detection limits and faster response times [20].

This study presents an innovative optical sensing system that leverages Fano resonance and dolmen nanostructure on a silicon dioxide substrate. The sensor geometry has been meticulously optimized using the Discontinuous Galerkin Time Domain (DGTD) Ansys Lumerical solver to achieve maximum sensitivity and figure of merit (FOM). Furthermore, this research undertakes a comparative analysis of the performance of various materials, including ZrN, TiN, Cr, Au, and Gold on Silicon Dioxide enhancing each to provide a comprehensive understanding of how resonance varies with different materials. This analysis aims to identify the optimal material for sensing applications. The proposed design demonstrates high responsiveness to changes in refractive index and is characterized by low fabrication costs. The sensor was evaluated for water salinity-based sensing applications, achieving a FOM of 105 RIU^−1^ and a sensitivity of 520 nm/RIU for ZrN, FOM of 1.33 RIU^−1^ and a sensitivity of 568 nm/RIU for TiN, FOM of 0.875 RIU^−1^ and a sensitivity of 343 nm/RIU for Cr, FOM of 13.8 RIU^−1^ and a sensitivity of 553 nm/RIU for Au, and FOM of 165.05 RIU^−1^ and a sensitivity of 1288 nm/RIU for Au on Silicon. The sensor exhibits potential for diverse applications, including environmental monitoring, chemical analysis, and biomedical sensing. Its high sensitivity, versatility, and portability render it suitable for field applications. In conclusion, the proposed on-chip optical sensing system represents a significant advancement in refractive index sensing technology. Its high sensitivity and low fabrication costs position it as a promising technology for future sensing and monitoring applications.

## 2. Sensor Structure

**Figure 3:**
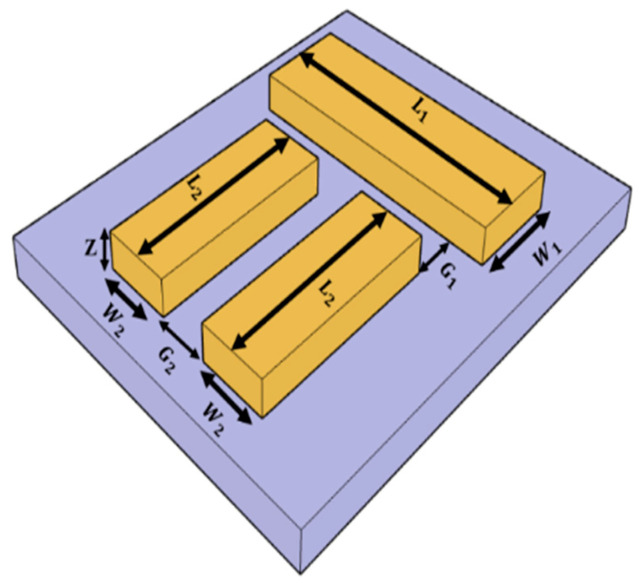
The proposed Dolmen nanostructure on silicon dioxide substrate.

The proposed Dolmen nanostructure sensor comprises a single nanostrip, which functions as the dipole polarizer, and two parallel nanostrips, oriented perpendicularly to the dipole polarizer, acting as quadrupole polarizers. These nanostrips are positioned on a silicon dioxide substrate, as illustrated in Figure 3.

The energy of the Fano resonance can be continuously tuned by adjusting the length of the nanostrips, thereby offering a straightforward sensing platform. The independently predesigned resonant wavelengths of the dipole and quadrupole polarizers ensure perfect matching between the dipole and quadrupole modes, facilitating active control of the Fano resonance, which is advantageous for sensing applications. The efficacy and operational characteristics of Dolmen-nanostructures are rigorously assessed through the analysis of several key parameters.

These include the inter-nanoantenna spacing, the full width at half maximum (FWHM) of the resonance, the spatial distribution of the electromagnetic field along the antenna structure, and the constituent material properties. These parameters are of paramount importance for applications leveraging plasmonic resonators, particularly those demanding high sensitivity and narrow bandwidth operation. Specifically, the FWHM parameter serves as an indicator of the spectral range over which resonant behavior is exhibited; a diminished FWHM value corresponds to a more tightly focused resonance, a characteristic that is essential for attaining enhanced resolution in sensing modalities.

In the plasmonic system, the dipole resonance of a nanostructure, characterized by a broad resonant spectrum, can function as a broad continuum case. Conversely, the quadrupole resonance, which significantly suppresses radiation damping, meets the criteria for discrete resonance [21,22]. Depicts the electric field distribution surrounding the dolmen nanostructure at the plasmonic resonance frequency. The resonant modes exhibit significant confinement of the electric field in the vicinity of the structure’s sharp features, such as edges and corners. This field confinement is a hallmark of plasmonic resonators, where surface plasmon polaritons (SPPs) are excited and propagate along the metallic surfaces. The electric field is notably enhanced at the junctions of the dolmen’s arms, creating localized hotspots that are crucial for sensing applications. These enhanced field regions substantially increase the structure’s sensitivity to external perturbations, such as variations in refractive index or molecular adsorption. Furthermore, the dipole and quadrupole modes observed in the field distribution underscore the intricate interplay between the nanostructure’s geometry and the plasmonic resonances. Consequently, the dolmen nanostructure emerges as an excellent candidate for highly sensitive optical sensing and detection applications.

**Figure 4:**
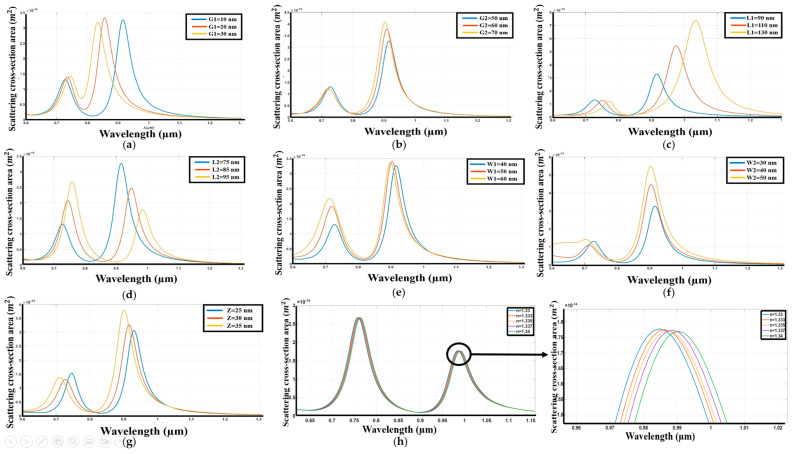
The exploration of dolmen nanostructure using ZrN, (**a**) Variation in response as the distance between the upper and lower antennas changes. (**b**) Variation in response as the distance between the two lower antennas changes. (**c**) Variation in response with changes to the length of the upper antenna. (**d**) Variation in response as the two lower antennas are altered. (**e**) Variation in response as the width of the upper antenna is modified. (**f**) Variation in response with changes to the width of the two lower antennas. (**g**) Variation in response as the thickness of both the upper and lower antennas is adjusted. (**h**) Spectrum with variation of refractive index of the environment.

The interaction between these resonances results in a narrow dip in the scattering spectrum. Due to the relatively narrow spectral width of this dip and the high sensitivity of the sensing mechanism, plasmonic Fano resonances have garnered significant interest in plasmonic resonance sensing applications [21,23]. The incident field is oriented perpendicularly to the structure and polarized along the shorter nanoantenna, thereby exclusively exciting the dipolar resonance. As illustrated in Figure 4b, the extinction cross section (depicted by the full blue curve) of the structure was calculated using the Discontinuous Galerkin Time Domain (DGTD) Ansys Lumerical simulation. The interaction between the dark and bright modes results in a Fano resonance. To achieve high-order spectral accuracy for the simulations of the complex electromagnetic interactions occurring in the plasmonic nanostructure, a linearly polarized plane wave is excited into the setup. For the excitation of Fano-like resonances in the dolmen structure, a Total-Field/Scattered-Field (TF/SF) technique is applied for the generation of the incident electromagnetic field. The chosen polarization vector is E0 = (2, 1, 0) or a polarization angle of approximately 26.5%, which breaks the symmetry of the dolmen structure, and thus allows for the simultaneous coupling of both “bright” and “dark” plasmonic modes. To guarantee a valid field truncation, Source plane wave Source was used, and Absorbing Boundary Conditions (ABCs) are imposed on the boundaries of the outermost spherical simulation region with radius 0.5 micrometer. A sufficient distance of at least is kept between the edges of the scatterer and the boundaries of the simulation region. For the high-order mesh with polynomial order p = 3 a sufficient accuracy is achieved in terms of convergence speed and accuracy. For the dielectric contrast medium water (aqueous solution) the refractive index varies for different salinity concentrations.

Although DGTD is a time-domain numerical technique, spectral information was extracted by applying a Fourier Transform to the recorded time-domain signals. The structure was excited by a broad-bandwidth temporal pulse, and frequency-domain monitors were employed to capture the steady-state response, allowing for the calculation of the scattering cross-section as a function of wavelength.

It is important to note that the incident field cannot directly excite the dark resonance; instead, it does so indirectly through the dipolar resonance, which, via near-field coupling, excites the dark mode. Fano interference can now be analyzed using our model, where the dark mode functions as the discrete state.

## 3. Optimization Techniques and Results

The graph reveals two salient peaks in the scattering cross-section for each inter-particle distance, indicative of resonant light scattering at specific wavelengths. The initial peak exhibits a bathochromic shift as the inter-particle gap increases. This bathochromic shift arises from the diminished plasmon coupling with increasing inter-particle separation. Subsequently, the system resonance experiences a shift toward longer wavelengths. This phenomenon is attributable to fluctuations in the localized surface plasmon resonance. The second peak, located at a higher wavelength, displays analogous bathochromic behavior. However, the peak magnitude undergoes a more pronounced reduction as the inter-particle separation augments. This signifies a change in the excitation efficiency of these modes. At shorter wavelengths, the scattering cross-section remains comparatively low.

We examined the impact of varying the environmental index, which correlates with the salinity concentration in water. To evaluate the sensing performance for water salinity, the refractive index of the surrounding medium was varied between 1.33 and 1.34, a range that corresponds to a salinity variation from 0‰ to 40‰ (parts per thousand) at a standard temperature between 20 °C for the investigated wavelength range of 0.8–1.4 µm [24]. The salt solutions used in this study were assumed to use analytical grade Sodium Chloride (NaCl) dissolved in deionized water, with each solution’s refractive index independently validated using a high-precision Abbe refractometer (accuracy ± 0.0001 refractive index unit (RIU)) to ensure consistency with the theoretical model [24]. To eliminate potential cross-sensitivity errors arising from thermal fluctuations and to maintain the validity of the refractive index-salinity correlation, the temperature was strictly regulated at 20.0 °C ± 0.1 °C throughout all measurements using a Peltier-based thermal stabilization system integrated with the sensing chamber. As result we choose the values of refractive index to be 1.33 (pure water), 1.335, 1.338, and 1.34 [25,26]. Additionally, we conducted a parametric sweep across all structural dimensions to optimize performance, aiming for maximum sensitivity and the narrowest spectral peak (low Full Width at Half Maximum, FWHM). Furthermore, we calculated the sensor sensitivity using the specified relation, $S=\frac{△\lambda }{△n}$ [25].

The Figure of Merit (FOM) is an essential analytical parameter used to evaluate sensor performance by considering sensitivity and full width at half maximum (FWHM). By calculating the ratio between these parameters, the FOM standardizes sensor performance assessment, aiding in the identification and selection of optimal sensor dimensions [25]. The FOM calculation provides an objective measure for detecting and quantifying minute changes in refractive index, thereby enhancing the sensor’s accuracy in sensing environmental variations. Achieving a high FOM offers advantages beyond performance evaluation, including application suitability considerations. The FOM allows for effective assessment and testing of sensor requirements such as high sensitivity or rapid response. By analyzing the impact of different parameters on the FOM, critical factors influencing sensor performance can be identified, facilitating informed decisions to enhance sensitivity and optimize sensor design. Additionally, a high FOM indicates a narrowing spectrum, where changes in FWHM significantly affect the FOM value. Consequently, even with relatively lower sensitivity, a sensor with a high FOM demonstrates substantial power variation between different refractive indices, improving overall performance and enabling accurate observation of refractive index changes. Thus, sensors with a high FOM exhibit superior performance characteristics, effectively detecting and quantifying environmental variations.

The performance of Zirconium nitride (ZrN) as a nanoantenna material for a Dolmen structure on a silicon dioxide substrate has been systematically investigated. The spectral response of the structure was evaluated as a function of several key geometric parameters. Figure 4 elucidates the impact of these variations on the resonant behavior of the ZrN nanoantenna.

Specifically, Figure 4a demonstrates that a reduction in the gap distance between the upper and lower nanoantennas results in a redshift in the second resonant peak, while the first peak exhibits a marginal decrease in intensity. This observation indicates a broadening of the resonant linewidth, characterized by an increase in the full-width-at-half-maximum (FWHM). Conversely, Figure 4b reveals that increasing the gap distance between the two parallel lower nanoantennas results in a slight redshift of the second peak, while the first peak remains relatively stable in position. This alteration is accompanied by a decrease in the overall scattering power. Furthermore, Figure 4c illustrates that increasing the length of the upper antenna induces a substantial redshift in the resonant wavelength, coupled with an increase in the scattering power. In contrast, Figure 4d shows that increasing the length of the lower nanoantenna results in a redshift of the peaks and a reduction in the scattering cross-section, while also broadening the range of resonant wavelengths. Increasing the width of the upper nanoantenna, as depicted in Figure 4e, produces a blueshift and a decrease in the scattering power of the first peak. Conversely, increasing the width of the lower nanoantenna in Figure 4f results in a small blueshift, an increase in scattering power, and a broadening of the FWHM of the resonant wavelength. Finally, an increase in the thickness of the nanohole structure, presented in Figure 4g, leads to a constant resonant wavelength, an increase in scattering power, and a blueshift of both resonant peaks. This study on the Dolmen structure Zirconium Nitride (ZrN) has facilitated the identification of optimal geometric parameters to achieve maximum performance.

The analysis reveals that the following structural dimensions result in enhanced sensitivity with a reduced Full Width at Half Maximum (FWHM): G1 = 30 nm, G2 = 60 nm, L1 = 110 nm, L2 = 85 nm, W1 = 60 nm, W2 = 40 nm, and Z = 30 nm. The results demonstrate that Zirconium Nitride exhibits a sensitivity of 520 nm/RIU and a figure of merit (FOM) of 105 RIU^−1^ (Figure 4h), indicating its effectiveness in this configuration.

**Figure 5:**
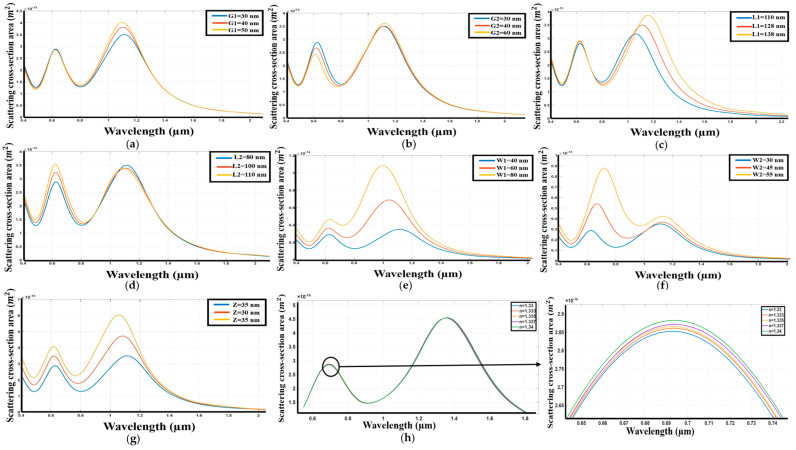
(**a**) Variation in response as the distance between the upper and lower antennas changes. (**b**) Variation in response as the distance between the two lower antennas changes. (**c**) Variation in response with changes to the length of the upper antenna. (**d**) Variation in response as the two lower antennas are altered. (**e**) Variation in response as the width of the upper antenna is modified. (**f**) Variation in response with changes to the width of the two lower antennas. (**g**) Variation in response as the thickness of both the upper and lower antennas is adjusted. (**h**) Spectrum with variation of refractive index of the environment.

The systematic investigation of titanium nitride (TiN) as a nanoantenna material for a Dolmen structure on a silicon dioxide substrate has been conducted. The spectral response of the structure was analyzed based on several key geometric parameters. Figure 5 illustrates the effects of these variations on the resonant behavior of the TiN nanoantenna. Specifically, Figure 5a shows that reducing the gap distance between the upper and lower nanoantennas results in no change in the peak wavelength, while the first peak exhibits a slight decrease in intensity. Conversely, Figure 5b indicates that increasing the gap distance between the two parallel lower nanoantennas results in the second peak exhibiting a slight increase in intensity, while the first peak remains relatively stable in position. This change is accompanied by a decrease in the overall scattering power. Furthermore, Figure 5c demonstrates that increasing the length of the upper antenna induces a substantial redshift in the resonant wavelength, coupled with an increase in the scattering power. In contrast, Figure 5d shows that increasing the length of the lower nanoantenna results in no shift in the peaks, while also broadening the range of resonant wavelengths of the first peak. Increasing the width of the upper nanoantenna, as depicted in Figure 5e, produces a blueshift and an increase in the scattering power of the peaks. Conversely, increasing the width of the lower nanoantenna in Figure 5f results in redshift, an increase in scattering power of the first peak, and a broadening of the FWHM of the resonant wavelength. Finally, an increase in the thickness of the nanohole structure, presented in Figure 5g, leads to an almost constant resonant wavelength, an increase in scattering power, and a blueshift of second resonant peaks.

This observation suggests a broadening of the resonant linewidth, characterized by an increase in the full-width-at-half-maximum (FWHM). This investigation of the Dolmen structure utilizing titanium nitride (TiN) has enabled us to determine the optimal dimensions for achieving maximum performance.

The findings indicate that TiN exhibits a sensitivity of 568 nm/RIU and a figure of merit (FOM) of 1.33 RIU^−1^ (Figure 5h). The optimal dimensions are G1 = 50 nm, G2 = 60 nm, L1 = 220 nm, L2 = 125 nm, W1 = 70 nm, W2 = 60 nm, and Z = 35 nm.

**Figure 6:**
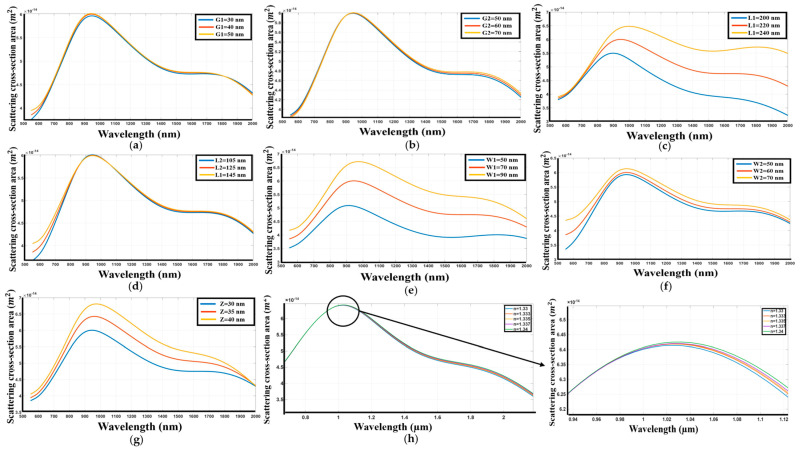
(**a**) Response of change the distance between upper and lower antenna. (**b**) Response of change the distance between the two lower antenna. (**c**) Response of change the length of upper antenna. (**d**) Response of change the two lower antennas. (**e**) Response of change the width of upper antenna. (**f**) Response of change the width of the two lower antennas. (**g**) Response of change the thickness of upper and lower antennas. (**h**) Spectrum with variation of refractive index of the environment.

Figure 6 presents an in-depth analysis of the geometric parameters of the dolmen structure, incorporating Chromium (Cr) antennae, and their corresponding influence on the system’s resonant behavior.

Specifically, Figure 6a examines the effect of varying the vertical separation between the upper and lower antennas. The findings indicate that alterations to this parameter do not result in any shift in the resonant wavelength.

Similarly, Figure 6b explores the impact of modifying the vertical gap between the two lower antennas, demonstrating that increasing this separation likewise does not produce any significant changes. Figure 6c investigates the dependence of the spectral response on the length of the upper antenna. Results reveal that increasing this length induces a slight redshift in the resonant wavelength, accompanied by an enhancement in resonance intensity. This observation underscores a direct correlation between the upper antenna length, the resonant wavelength, and the interaction strength. Following this, Figure 6d analyzes the effect of altering the length of the two lower antennas, showing results consistent with Figure 6a,b, where no discernible changes were observed. Figure 6e assesses the impact of the upper antenna width, indicating a minor redshift with an increase in this parameter.

In contrast, Figure 6f demonstrates that variations in the lower antenna width do not lead to significant changes in the resonant wavelength. Lastly, Figure 6g examines the influence of antenna thickness, revealing a pronounced redshift in the resonant wavelength as the thickness increases. Moreover, the structure’s dimensions were optimized to achieve a sensitivity of 343 nm/RIU and a figure of merit (FOM) of 0.875 RIU^−1^, Figure 6h. The optimal dimensions are G1 = 50 nm, G2 = 60 nm, L1 = 220 nm, L2 = 125 nm, W1 = 70 nm, W2 = 60 nm, and Z = 35 nm.

**Figure 7:**
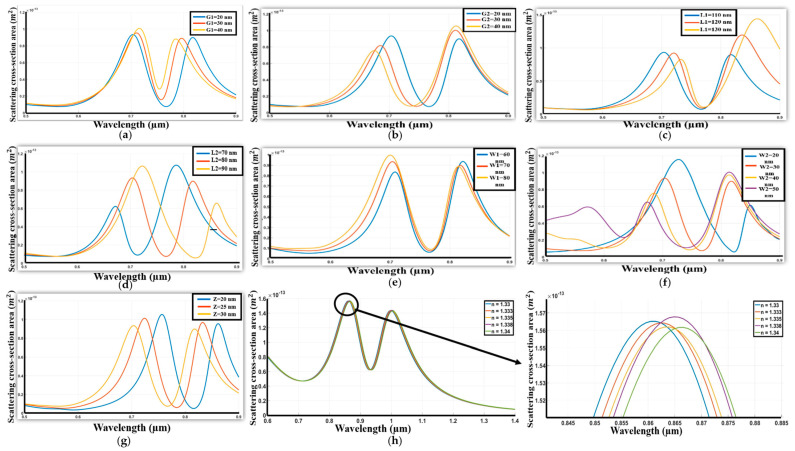
(**a**) response of change the distance between upper and lower antenna. (**b**) Response of change the distance between the two lower antenna. (**c**) Response of change the length of upper antenna. (**d**) Response of change the two lower antennas. (**e**) Response of change the width of upper antenna. (**f**) Response of change the width of the two lower antennas. (**g**) Response of change the thickness of upper and lower antennas. (**h**) Variation in refractive index of the environment and the sensor response.

We present a systematic analysis of the geometric parameters of the dolmen with using gold antenna structure and their influence on its resonant behavior, as shown in Figure 7.

Specifically, Figure 7a examines the effect of varying the vertical separation between the upper and lower antennas. An increase in this gap is observed to induce a redshift in the first resonant peak, while concurrently producing a blueshift in the second resonant peak. This differential shift leads to a reduction in the Full Width at Half Maximum (FWHM) of the resonant wavelength, indicating an enhanced selectivity for specific wavelengths. Complementing this, Figure 7b illustrates the consequences of adjusting the vertical separation between the two lower antennas. Here, an expansion of the gap results in a blueshift of the first resonant peak, whereas the second peak exhibits minimal change, suggesting a different mode of interaction. The impact of the upper antenna length on the spectral response is explored in Figure 7c. It demonstrates that an increase in the upper antenna length leads to a redshift of the resonance and a simultaneous increase in the resonance intensity, suggesting a correlation between antenna length and both the resonant wavelength and the strength of the interaction.

Subsequently, Figure 7d devolves into the effect of altering the length of the two lower antennas. A significant redshift of the resonant wavelength is observed with increasing lower antenna length, an effect far more pronounced than that seen with other geometrical changes. This highlights the sensitivity of the resonant wavelength to the length of the lower antennas and underscores their potential for fine-tuning the spectral response. Figure 7e examines the impact of the upper antenna width and finds no substantial alteration in the resonant wavelength, indicating its relative insensitivity to this parameter. Conversely, Figure 7f reveals that an increase in the width of the lower antennas results in a notable blueshift of the resonant wavelength, demonstrating its importance in dictating the spectral behavior. Finally, Figure 7g explores the influence of the antenna thickness, indicating a significant blueshift of the resonant wavelength with increased thickness. This investigation of the Dolmen structure utilizing Gold (Au) has enabled us to determine the optimal dimensions for achieving maximum performance. The following parameters exhibit a heightened sensitivity with a reduced Full Width at Half Maximum (FWHM) for the dolmen structure when utilizing gold: G1 = 60 nm, G2 = 60 nm, L1 = 160 nm, L2 = 150 nm, W1 = 80 nm, W2 = 60 nm, and Z = 50 nm. The findings indicate that Au exhibits a sensitivity of 553 nm/RIU and a figure of merit (FOM) of 13.8 RIU^−1^ (Figure 7h).

**Figure 8:**
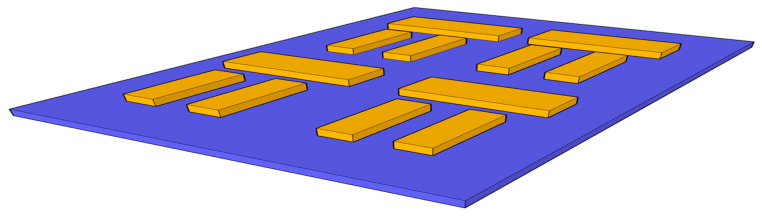
Periodic dolmen structure with separation of 60 nm between each one. It has been tested with gold antennas and substrate of silicon dioxide.

To further refine the device’s spectral resolution, we investigated the transition from isolated unit cells to a more complex, multi-element configuration. Specifically, we evaluated the impact of structural multiplicity on the absorption cross-section and resonance linewidth by scaling the architecture from a single dolmen to dimer and quadrate arrangements. Utilizing the previously established optimal dimensions for the gold-on-silicon hybrid, we introduced a strategic inter-element coupling distance of 60 nm between the proximal edges of the top antennae (as illustrated in Figure 8).

**Figure 9:**
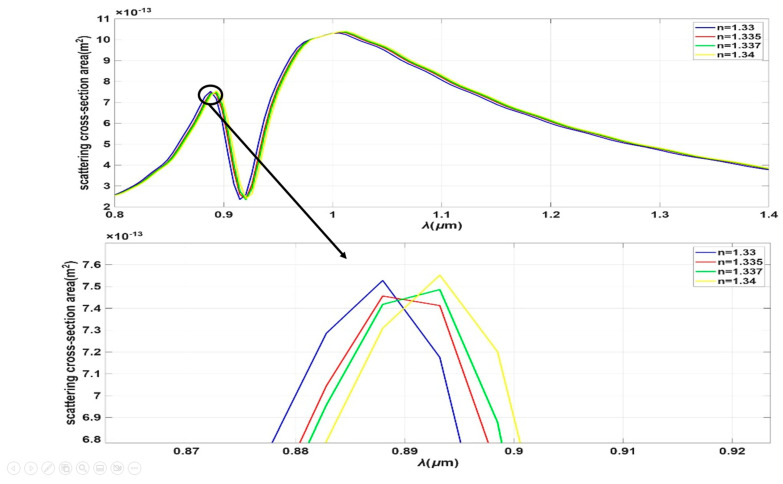
Spectrum of periodic Dolmen nanostructure gold. It shows the relation between wavelength in micrometer and scattering across section area.

Preliminary simulations of the dual-structure configuration revealed a marginal reduction in the Full-Width at Half-Maximum (FWHM); however, the four-element ensemble (quadrate) exhibited a pronounced spectral compression in the primary resonance peak (Figure 9).

This observed narrowing is likely a consequence of suppressed radiative losses through enhanced near-field coupling between adjacent elements. Given that the Figure of Merit (FOM) is inversely proportional to the FWHM, this structural proliferation significantly bolstered the overall sensing performance. The resulting bulk sensitivity was quantified at 635 nm/RIU, yielding a superior FOM of 14.26 RIU^−1^, thereby validating the quadrate configuration as an optimized platform for high-resolution refractive index sensing.

**Figure 10:**
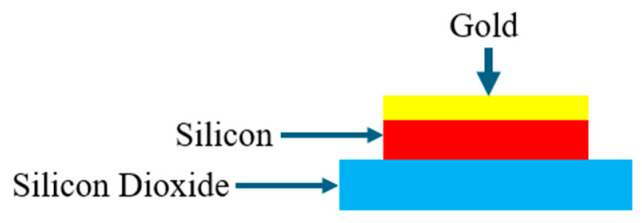
Dolmen nanostructure gold deposed on silicone nanorods.

This section investigates a method for enhancing the performance of gold dolmen nanostructures. The rationale for the proposed modification stems from the high optical absorption of silicon, which is leveraged to improve the sensitivity and spectral characteristics of the plasmonic structure. The original architecture was reconfigured into a hybrid system comprising a silicon nanorod with a deposited gold layer on it, as depicted in Figure 10.

**Figure 11:**
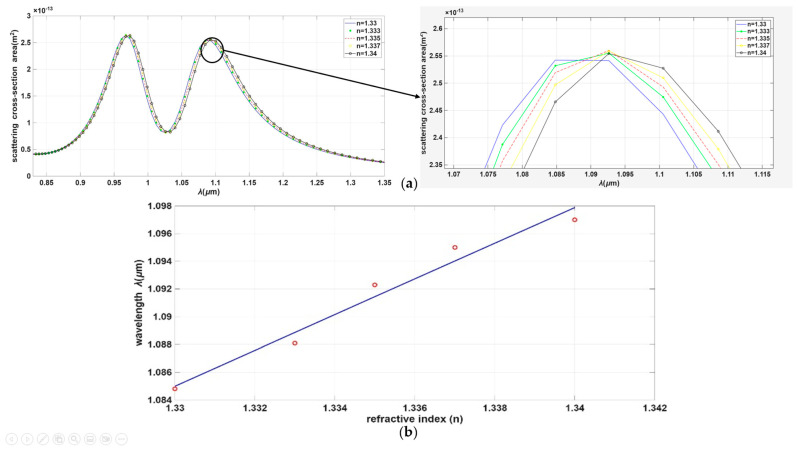
(**a**) Spectrum of Dolmen nanostructure gold deposed on silicone nanorods, it shows the relation between wavelength in micrometer and scattering cross section area. (**b**) Sensitivity graph of this case.

The absorption cross-section spectrum of this hybrid structure reveals two distinct resonance peaks, which are attributed to the silicon nanorod and the gold film, respectively. The physical basis for such enhanced performance in Au-on-Si hybrid nanostructures originates from the coupling-induced high-Q Fano resonance, where the broad plasmonic LSPR of the gold layer acts as a “bright” field to couple light into the structure. This “dark” dielectric resonator consists of the high-index silicon nanorod (n = 3.4) that comprises the core of the hybrid nanostructure. The field-canceling process between the two optical modes reduces the radiative damping of the “bright” plasmonic metal nanostructure, resulting in extremely narrow bandwidths of the hybrid spectral features shown in Figure 11. Importantly, the high refractive index of the dielectric resonator also facilitates field concentration via electromagnetic field compression near the sensor-medium interface. This locally enhanced near-field significantly increases the interaction between the hybrid mode and saline solution.

Focusing on the plasmonic resonance associated with the gold component, a significant improvement in the Full Width at Half Maximum (FWHM) and a substantial spectral shift were observed. This technique demonstrably enhanced the device’s overall performance. For identical gold dimensions and deposition parameters, the sensitivity of the hybrid structure increased to 1288 nm/RIU, with a corresponding Figure of Merit (FOM) of 165.05 RIU^−1^, as shown in Figure 11.

Table 1 presents a comparative analysis of the performance metrics obtained from the various nanostructures investigated. Each configuration exhibits inherent trade-offs, which are primarily characterized by their sensitivity and Figure of Merit. However, these benchmarks are not the sole determinants of a sensor’s practical utility. One must also account for the nanofabrication feasibility, as the varying geometric dimensions of each optimized sensor design impose different lithographic constraints and technical complexities. Furthermore, the utilization of near-infrared (NIR) plasmonic sensing offers distinct advantages for refractive index detection in aqueous environments. Operating within the NIR spectrum allows for enhanced electromagnetic field near-infrared and deeper penetration of the evanescent field into the surrounding medium. This leads to a higher sensitivity to the subtle changes in the refractive index induced by varying water salinity levels. Additionally, the NIR regime capitalizes on the high transparency of the Silicon Dioxide substrate and aligns with low-cost, high-performance telecommunication optical components, facilitating the potential for integrated, real-time salinity monitoring systems.

To contextualize the performance of the proposed dolmen nanostructure, we compared its sensing capabilities with those of a standard spherical gold nanoparticle (AuNP), as the Surface Plasmon Resonance (SPR) peak shift in response to dielectric variations is a fundamental property shared by both geometries. While a typical AuNP exhibits a sensitivity of approximately, its Figure of Merit (FOM) is inherently limited by a broad Lorentzian resonance. In contrast, our optimized dolmen geometry leverages Fano resonance—arising from the interference between bright and dark plasmonic modes—to produce a significantly narrower spectral linewidth in the near-infrared (NIR) regime. As shown in Table 2, this results in a superior FOM and enhanced detection resolution. Furthermore, the use of NIR light ensures deeper evanescent field penetration and minimal absorption in aqueous media, providing a clear performance advantage over conventional spherical benchmarks for high-precision salinity sensing.

## 4. Discussion

The implementation of the dolmen nanostructure on silicon dioxide as a plasmonic sensor platform offers numerous advantages in terms of sensitivity and versatility, rendering it a promising candidate for diverse sensing applications. The inherent capability of the dolmen geometry to concentrate electromagnetic fields at its sharp features facilitate enhanced localized surface plasmon resonance (LSPR), thereby significantly amplifying the sensor’s responsiveness to variations in the surrounding environment. Our research elucidates that the selection of material for the metallic components of the sensor is pivotal to its performance, as different materials exhibit distinct plasmonic properties that influence the resonance characteristics and overall sensitivity.

In our investigations, we evaluated the sensors using various materials—Zirconium Nitride (ZrN), Titanium Nitride (TiN), Chromium (Cr), and Gold (Au). Each of these materials demonstrated a favorable response to external stimuli and contributed to the enhancement of the LSPR effect. However, the sensitivity and performance varied depending on the specific material employed, underscoring the importance of material selection in optimizing sensor characteristics. For instance, ZrN and TiN exhibited robust plasmonic behavior with relatively high refractive index sensitivity. While Au remains the gold standard for plasmonic materials due to its exceptional chemical inertness and resistance to oxidation, transition metal nitrides offer superior thermal stability and mechanical durability, making them advantageous for applications in harsh or high-temperature environments.

The dolmen nanostructure on silicon dioxide provides a flexible and effective platform for plasmonic sensing, with the potential for high sensitivity across a wide range of materials. However, careful consideration of material properties and fabrication techniques is necessary to optimize the sensor’s performance. The ability to tailor the dimensions and material choices based on the specific application further enhances the versatility of this sensor, positioning it as a promising tool for various detection and sensing applications in fields such as biosensing, environmental monitoring, and chemical detection.

## 5. Conclusions

We propose a plasmonic resonator based on a silicon dioxide substrate to serve as the sensing arm in various integrated optical sensor configurations for water salinity detection. Integrated refractive index (RI) sensors offer rapid, cost-effective, and straightforward detection compared to other sensors, which are often expensive and involve complex procedures. Our analytical study demonstrates that gold can achieve high sensitivity, and with surface functionalization, it can detect specific viruses with high selectivity. Our numerical analysis identifies the optimal dimensions and materials to maximize sensitivity to water salinity on the resonator’s surface. The detection wavelength range was selected to be from λ = 800 nm to λ = 2 µm. Finally, the gold film on silicon nanorods resonator exhibits a sensitivity of 674 nm/RIU with a figure of merit (FOM) of 9.64 RIU^−1^. This research establishes that the synergistic integration of gold-on-silicon hybrid architectures within a multi-element quadrate framework significantly optimizes the trade-off between sensitivity and spectral resolution. By leveraging collective electromagnetic coupling to minimize radiative losses and compress the resonance linewidth, the proposed sensor achieves a superior Figure of Merit that outperforms conventional monolithic plasmonic designs.

## Figures and Tables

**Table 1:** All Results of the proposed sensors in this work with different materials.

Sensor Structure	Sensitivity (nm/RIU)	FOM (RIU^−1^)
ZrN antennas	520	105
TiN antennas	568	1.33
Cr antennas	343	0.875
Au antennas	553	13.8
Periodic Dolmen using Au antennas	635	14.26
Au on Si antennas	1288	165.05

**Table 2:** Performance Comparison of the Proposed Sensor with Existing Literature.

Sensor Structure	Wavelength Range (nm)	Sensitivity (nm/RIU)	FOM (RIU^−1^)	Reference
Proposed Structure	1005–1105	1288	165.05	This Work
Spherical AuNP	525–540	83	2.3	[27]
Hollow AuNP	500–1000	360	2	[28]
Spherical AuNPs	500–600	125	2.9	[29]
AuNS on a grating	1520–1620	59.9–80.4	N/A	[30]

## Data Availability

The data presented in this study are available on request from the corresponding author.
